# LKB1 Loss Sensitizes Lung Tumor Spheres to Mitomet‐Induced Ferroptosis, and These Effects are Enhanced by mTOR Inhibition

**DOI:** 10.1002/mc.70129

**Published:** 2026-05-15

**Authors:** Xueqing Liang, Ali Nakhi, Peter I. Dosa, Gunda I. Georg, Fekadu Kassie

**Affiliations:** ^1^ Masonic Cancer Center University of Minnesota Minneapolis MN USA; ^2^ Department of Medicinal Chemistry and Institute for Therapeutics Discovery and Development, College of Pharmacy University of Minnesota Minneapolis MN USA; ^3^ College of Veterinary Medicine University of Minnesota Saint Paul MN USA

**Keywords:** cancer stem cells, ferroptosis, LKB1 loss, mitochondria, Mitomet, non‐small cell lung cancer, spheres

## Abstract

Owing to their robust antioxidant defense mechanisms, cancer stem‐like cells (CSCs) maintain a low level of oxidative stress, which is crucial for preserving stemness and pluripotency. Therefore, agents that either directly generate reactive oxygen species (ROS) or inhibit the antioxidant defense systems can selectively induce oxidative cell death in CSCs. Loss of the tumor suppressor gene LKB1 makes CSCs more vulnerable to oxidative damage, as these cells cannot sense energy stress. In the present study, LKB1 wild‐type (WT) and LKB1 mutant isogenic non‐small cell lung cancer (NSCLC) cells were grown in sphere culture media, which enriches the CSC population, and treated with mitomet, an analog of the antidiabetic drug metformin. Subsequently, effects on self‐renewal of spheres, mitochondrial membrane potential (MMP), ATP synthesis, expression of self‐renewal‐, cell proliferation/survival‐, redox metabolism‐, and lipid synthesis‐related proteins, mitochondrial ROS (mROS), lipid peroxidation, and cell proliferation/survival were determined. Mitomet differentially increased mROS in LKB1 mutant tumor spheres, thereby suppressing levels of MMP, ATP synthesis, GPX4, and phospho‐ACC, which then culminated in increased lipid peroxidation and cell death. Mitomet‐induced lipid peroxidation and cell death were reversed by liproxstatin, a potent inhibitor of ferroptosis, indicating that mitomet‐induced cell death was mediated via ferroptosis. Interestingly, Torin‐1, an mTOR inhibitor, significantly potentiated lipid peroxidation and ferroptosis induced by mitomet. Our findings establish the potential of mitomet, especially when combined with mTOR inhibitors, for the prevention and treatment of LKB1 mutant lung cancer by targeting CSCs.

## 1 Introduction

Mutations in the Kirsten rat sarcoma viral oncogene homolog (m*KRAS*) occur in approximately half of non‐small cell lung cancer patients [1, 2], and about 8–31% of Kras mutant lung tumors exhibit co‐occurring mutations in the serine/threonine kinase 11 gene (*LKB1/STK11* [3, 4], KL tumors). KL tumors are highly aggressive and do not respond to any of the currently existing therapies. However, the underlying mechanisms responsible for the aggressiveness of these tumors remain elusive. The presence of cancer stem‐like cells (CSCs), considered to be putative tumor‐initiating cells, in solid tumors has been confirmed in several malignancies, and these cells, in addition to their ability for self‐renewal and differentiation, are highly tumorigenic and resistant to chemotherapeutics. Given the shared properties of KL tumors and CSCs, it is possible that CSCs play a role in many of the clinicopathological features of KL tumors. Consistent with this, KL lung tumors have been found to harbor stronger stemness and display significant up‐regulation of Nanog expression, in contrast to lung tumors exhibiting alteration with Kras alone or with co‐occurring Kras and p53 mutation [5].

An increasing body of evidence demonstrates that KL lung cancers are heavily reliant on oxidative phosphorylation (OXPHOS) for energy production [6]; thus, these cancers are particularly sensitive to OXPHOS inhibition. Similarly, although it has been believed for a long time that CSCs favor anaerobic glycolysis over OXPHOS for energy synthesis, emerging evidence indicates that OXPHOS is the preferred energy source for CSCs. In fact, CSCs from lung cancer [7], glioblastoma [8], pancreatic ductal adenocarcinoma (PDAC) [9], and leukemia [10] are known to use OXPHOS as the preferred energy production process. Therefore, OXPHOS is an “Achilles’ heel” for CSCs because their survival and high energy demands are met through OXPHOS, making them vulnerable to its inhibition [11]. Moreover, inhibition of OXPHOS can paradoxically increase the generation of reactive oxygen species (ROS) by causing electrons to “leak” from the electron transport chain (ETC) and react with oxygen. Owing to the low level of ROS in CSCs [12], resulting from their powerful scavenger antioxidative enzyme system [13, 14], these cells could be particularly sensitive to ROS‐generating agents and drugs that target the ROS defense system of CSCs.

LKB1 is a key upstream regulator of the AMP‐activated protein kinase (AMPK), which is activated upon energy stress and restores energy balance by promoting ATP‐generating catabolic processes and inhibiting ATP‐consuming anabolic processes [15]. In line with this, energy‐stress‐induced AMPK activation in LKB1 WT NSCLC cells has been shown to inhibit ferroptosis, an iron‐dependent regulated cell death caused by massive lipid peroxidation‐mediated membrane damage [16, 17], whereas LKB1 loss and the resulting reduced AMPK activity make these cells more susceptible to lipid peroxidation and ferroptosis. These findings suggest that, owing to their inability to sense and respond to energy loss, treatment of NSCLC cells lacking LKB1 with OXPHOS inhibitors may lead to a “synthetic lethal‐like” effect and enhance ferroptosis. However, whether energy stress inducers enhance ferroptosis in NSCLC cells with LKB1 loss is unknown.

In the present study, we used LKB1‐deficient/proficient isogenic tumor spheres, non‐adherent colonies of cells derived from a single cancer stem cell (CSC [18]), to determine the impact of LKB1 loss on self‐renewal, expression of stemness‐, cell proliferation/growth‐ and redox‐related proteins, level of mitochondrial ROS (mROS), and lipid peroxidation. Moreover, we assessed whether LKB1 loss modulates the sensitivity of NSCLC tumor spheres towards mitomet, a mitochondria‐targeted analog of metformin and a potent inhibitor of OXPHOS [19, 20, 21]. Levels of CD44, nanog, SOX2, and CD133 were not consistently different between LKB1 WT and LKB1 mutant spheres. On the other hand, expression of p‐AMPK, phospho‐4E‐BP1, phospho‐GSK3β, phospho‐ACC, and GPX4 was lower in LKB1 mutant spheres than in LKB1 WT spheres, whereas the level of mitochondrial ROS (mROS) was higher in LKB1 mutant spheres, particularly in LKB1 mutant H2030 spheres. Treatment of LKB1 WT and LKB1 mutant tumor spheres with mitomet differentially reduced self‐renewal, mitochondrial membrane potential, ATP levels, and expression of phospho‐ACC and GPX4 in LKB1‐deficient tumor spheres, whereas mROS and lipid peroxidation were increased. Silencing of GPX4 reduced the proliferation of spheres, but the effect was less than that of mitomet. Co‐treatment of tumor spheres with mitomet and liproxstatin‐1 (Lipro‐1), an antioxidant drug that inhibits ferroptosis by targeting lipid peroxidation and restoring the expression of GPX4, significantly attenuated mitomet‐induced lipid peroxidation and cytotoxicity, indicating that mitomet‐induced cell death is mediated via ferroptosis. Interestingly, the combination of mitomet and the mTOR inhibitor Torin‐1 selectively potentiated mitomet‐induced toxicity and lipid peroxidation in LKB1 mutant tumor spheres, which were attenuated by Lipro‐1. Overall, the present study shows that the OXPHOS inhibitor mitomet preferentially induces ferroptosis in LKB1‐deficient tumor spheres by promoting the generation of mROS and lipid peroxidation, and these effects are enhanced by targeting the PI3K/Akt/mTOR pathway, suggesting the potential of simultaneous targeting of OXPHOS and the mTOR pathway for the treatment of LKB1‐deficient NSCLC.

## 2 Materials and Methods

### 2.1 Chemicals and Cells

Mitomet was synthesized from metformin as described previously [19]. Liproxstain‐1 (Lipro‐1) and Torin‐1 were purchased from MedChemExpress LLC. Non‐small cell lung cancer (NSCLC) cell lines A549 and H2030 were purchased from ATCC. Both cell lines were tested for mycoplasma infection and authenticated by the short tandem repeat method at MD Anderson's Cell Line Core Facility in March 2023. Establishment of A549 and H2030 cells expressing LKB1 was described previously [19]. Briefly, LKB1 coding sequences (Sino Biological) were PCR amplified and cloned into the piggyBac transposon vector system (containing aCMV promoter and a NEO selection cassette) via the Gateway cloning method. Subsequently, LKB1 mutant A549 and H2030 cells were transfected with WT LKB1‐piggyBac expression vector and transposase plasmid using Lipofectamine 3000 reagent (Invitrogen). After 3 days of transfection, single‐cell clones resistant to G‐418 (200 μg/mL) were selected and LKB1 expression was confirmed by western blot analysis. A549 LKB1 WT/mutant cells were maintained in 10% FBS, F12K media (Corning), whereas H2030 LKB1 WT/mutant cells were maintained in 10% FBS, ATCC RPMI 1640 media (American Type Culture Collection). Tumor spheres were generated by culturing A549 and H2030 LKB1 WT/mutant cells in tumor sphere media containing Dulbecco's Modified Eagle Medium (DMEM)/F12 (Sigma), supplemented with 20 ng/mL rhEGF (R&D), 20 ng/mL human bFGF (R&D), 0.4% BSA (Sigma), 1% Insulin–Transferrin–Selenium (Invitrogen) with (for cell culture) or without 1% Pen/Strep (for siRNA transfection). On day 3 of cell culture, images of tumor spheres were acquired on an Olympus IX71 inverted fluorescence microscope with an Olympus DP12 digital microscope camera at 100× magnification.

### 2.2 Extreme Limiting Dilution Analysis (ELDA) to Determine the Self‐Renewal of Tumor Spheres

NSCLC cells were seeded at the densities of 20 cells, 10 cells, 5 cells, and 1 cell per well in 200 µL of tumor sphere media, with 12 replicates/group, and cultured with/without 2.5 µM mitomet or 10 µM IACS‐010759 in a 96‐well flat‐bottom ultra‐low attachment plate (Corning). On day 7 of culture, any wells that contained one or more spheres≥ 20 µm in diameter were scored. Data were analyzed as described by Hu et al. [22] using the ELDA software at https://bioinf.wehi.edu.au/software/elda/.

### 2.3 Detection of CD133 Expression, Mitochondrial ROS, and Lipid Peroxidation Levels By Flow Cytometry

To determine CD133 expression level in adherent cells and tumor spheres, NSCLC cells (5×10^4^ cells/well) were cultured in a 24‐well flat‐bottom ultra‐low attachment plate in tumor sphere media or cultured in a 24‐well flat‐bottom plate in regular culture media for 3 days. Single cells were stained with anti‐CD133 APC antibody or mouse IgG isotype control (BD Biosciences) and mean fluorescence intensity (MFI) was analyzed by flow cytometry. To determine the level of mitochondrial reactive oxygen species (mROS), NSCLC cells (5×10^4^ cells/well) were cultured in tumor sphere media without or with mitomet (2.5, 5 or 10 µM) in 24‐well flat bottom ultra‐low attachment plate or cultured in 24‐well flat bottom plate in regular culture media for 3 days, single cells were stained with 5 µM MitoSox Red Mitochondrial Superoxide Indicator (Thermo Fisher Scientific) at 37°C for 30 min, and MFI was analyzed by flow cytometry at 396/610 nm excitation and emission wavelengths.

To determine the level of lipid peroxidation, cells were cultured with or without mitomet (10 µM) for 5 days as described above. Subsequently, single suspension cells were stained with 2 µM BODIPY 581/591 C11 at 37°C for 30 min and analyzed by flow cytometry at different excitation and emission wavelengths (reduced state at the 581/591 nm and oxidation state at 488/510 nm). Lipid peroxidation was determined by the MFI ratio of oxidation state to the reduced state.

### 2.4 Transfection of Tumor Spheres with GPX4 siRNA

NSCLC cells were cultured at 3×10^4^ cells/well in 500 µL sphere media in a 24‐well flat‐bottom

ultra‐low attachment plate overnight, then the cells were transfected with 15 µM MISSION®

esiRNA targeting human GPX4 or siRNA Universal Negative Control (Millipore Sigma) using Lipofectamine RNAiMax reagent (Thermo Fisher Scientific) per the manufacturer's protocol. Then, 1 ml tumor sphere media was added to the cells the next day, and the cells were cultured for another 24 h (for immunoblot analysis of GPX4 protein) or 5 days (for cell viability assays).

### 2.5 Cell Viability Determination Using Trypan Blue Exclusion, TMRE, Propidium Iodide (PI) Staining, and ATP Quantitation Assays

To assess the time‐dependent cytotoxic effects of mitomet towards tumor spheres, NSCLC cells (7×10^4^ cells/well) were cultured in tumor sphere media with or without 10 µM mitomet in a 24‐well flat‐bottom ultra‐low attachment plate up to 5 days. To determine the cytotoxic effects of GPX4 siRNA, A549 LKB1 WT/LKB1 mutant cells (3×10^4^ cells/well) were cultured in a 24‐well flat‐bottom ultra‐low attachment plate in tumor sphere media overnight and transfected with control siRNA or GPX4 siRNA overnight. On day 5, tumor spheres were harvested, dissociated into single cells with Trypsin‐EDTA, and the cell number was determined by trypan blue exclusion assay. For assays using TMRE and PI, cells were cultured in sphere media for 5 days without or with 10 µM mitomet, stained with 200 nM tetramethyl‐rhodamine ethyl ester (TMRE, Sigma) at 37°C for 10 min or 5 µL propidium iodide (PI, BD Bioscience) for 15 min at room temperature, and analyzed by flow cytometry. Data were presented as the percentage of TMRE‐positive viable cells or PI‐positive dead cells.

For the ATP‐based cell viability assay, NSCLC cells were cultured at 5×10^3^ cells/well in 100 µL tumor sphere media in a 96‐well U‐bottom ultra‐low attachment plate (S‐BIO), and cells were treated with mitomet (0, 2.5, 5, and 10 µM) for up to 5 days. Subsequently, 100 µL of CellTiter‐Glo 3D Reagent (Promega) was added to each well on day 5 of cell culture to determine the number of viable cells in 3D culture by quantifying the ATP present, an indicator of metabolically active cells. Luminescent (RLU) signal intensity was determined by Tecan Infinite M200 Pro Multimode Microplate Reader (Tecan Group Ltd). The RLU intensity of each culture condition was compared to that of the media. Images of tumor spheres in cultures were acquired on an Olympus DP12 digital microscope at 100× magnification.

### 2.6 Lipro‐1 or Torin‐1 Blocking

To determine if the antioxidant agent Lipro‐1 blocks mitomet‐induced lipid peroxidation and ferroptosis, LKB1 mutant/WT A549 or H2030 tumorspheres (5×10^4^ cells/well) were pretreated with 1 μM Lipro‐1 for 2 h in a 24‐well flat‐bottom ultra‐low attachment plate and cultured in tumor sphere media with 10 μM mitomet. To determine if Torin‐1 can potentiate mitomet‐induced lipid peroxidation and ferroptosis, LKB1 mutant/WT A549 or H2030 tumorspheres (5×10^4^ cells/well) were cultured in a 24‐well flat‐bottom ultra‐low attachment plate in tumor sphere media with different doses of Torin‐1 (0.25 μM, 0.5 μM, and 1 μM) with or without 2.5 μM mitomet. In some experiments, cells were pretreated with 1 μM Lipro‐1 for 2 h, and cultured in a 24‐well flat‐bottom ultra‐low attachment plate in tumor sphere media with or without 1 μM Torin‐1 and/or 2.5 μM mitomet. Cell viability was determined on day three by trypan blue exclusion and PI staining with flow cytometry. The level of lipid peroxidation was determined on day 5 by flow cytometry after BODIPY 581/591 C11 staining.

### 2.7 Western Blot Assay

Whole cell protein lysates were prepared from adherent cells or tumor spheres lysed with RIPA lysis buffer. An equal amount of protein was fractionated on 4–12% NuPAGE^TM^ Bis‐Tris mini Gel (Invitrogen) and transferred onto PVDF membrane (Bio‐Rad). After blocking with 5% non‐fat milk, the membrane was probed with designated primary antibodies for Nanog, Nrf2, ACSL4, LKB1, pSer473 AKT, AKT, pSer9 GSK3β, GAPDH (Santa Cruz Biotechnology), SOX2, GPX4, xCT, pSer79 ACC, ACC, SCD1, pThr172 AMPK, AMPK, pSer37/46 4E‐BP1, 4E‐BP1, pSer235/236 S6, S6, GSK3β (Cell Signaling Technology). Subsequently, membranes were probed with horseradish peroxidase conjugated secondary antibody (Santa Cruz Biotechnology), visualized by ECL Western Blotting Substrate (Thermo Fisher Scientific Inc). The blots were imaged by the iBright 1500 imaging system (Thermo Fisher Scientific Inc), and the protein levels were quantified using the iBright Analysis Software in the imaging system. GAPDH was used as a loading control. For each protein, at least three western assays were carried out.

### 2.8 Detection of Intracellular GSH

A549 LKB1 mutant/WT cells were cultured in F12K media containing 10% FBS with or without 10 µM mitomet in a 6‐well plate, or cultured in tumor sphere media with or without 10 µM mitomet in a 6‐well ultra‐low attachment plate. Cells were harvested on day 3 of culture. Total GSH and GSSG (oxidized GSH) were measured by Glutathione Colorimetric Detection Kit (Invitrogen) per the protocol of the manufacturer.

### 2.9 Data Analysis

All data were reported as mean ± standard deviation (SD) of triplicate determinations. Statistical analysis of the data was performed by two‐way ANOVA or one‐way ANOVA, and *P* values less than 0.05 or 0.01 were considered statistically significant. All analyses were conducted using GraphPad Prism 9 software (GraphPad).

## 3 Results

### 3.1 Effects of LKB1 Loss on the Self‐Renewal Capacity and Expression of Cell Proliferation‐, Redox Balance‐ and Lipid Synthesis‐Related Proteins in NSCLC Tumor Spheres

**Figure 1:**
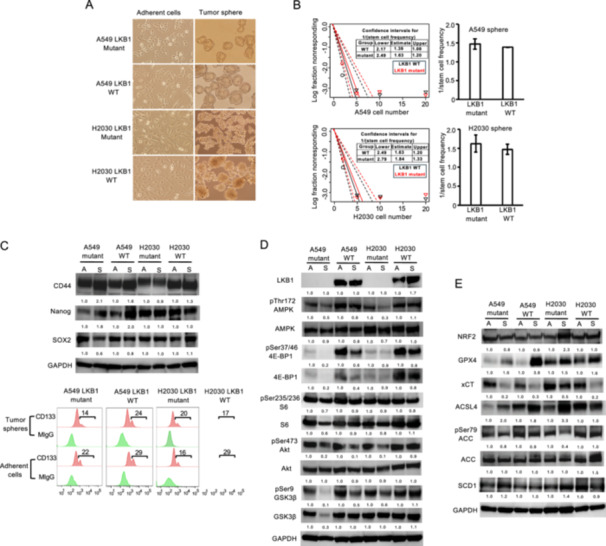
Comparison of cancer stem cell‐related characteristics between LKB1 WT and LKB1 mutant NSCLC tumor spheres. (A) Morphology of LKB1 WT and LKB1 mutant A549 and H2030 cells cultured under adherent (two‐dimensional) and sphere (three‐dimensional) conditions. (B) Extreme limiting dilution analysis (ELDA) for the assessment of the self‐renewal of LKB1 WT and LKB1 mutant tumor spheres. In the log‐fraction plots, the y‐axis “log fraction nonresponding” indicates the frequency of cells incapable of forming clonal spheres, whereas the x‐axis “cell number” indicates the number of cells per well. The slope of the line is the log‐active cell fraction, and dotted lines give the 95% confidence interval. Bar graphs show mean ± SD of the frequency of cancer stem cells (CSCs) calculated from three experiments, and 1/stem cell frequency refers to the reciprocal of the estimated CSC frequency or the number of cells in the population that contains, on average, one CSC. (C–E) Representative Western blot (C–E) and flow cytometry (C) results showing level of expression of stemness (C)‐, cell proliferation and growth‐ (D) and redox metabolism‐ (E) related proteins in adherent LKB1 WT and LKB1 mutant A549 and H2030 cells and their respective tumor spheres. “A” and “S” stand for adherent cells and tumor spheres, respectively. The assays were performed three times; the data shown are representative results from 1 of 3 independent experiments. In Western blot assays, protein expression was normalized to the expression of GAPDH, and the relative expression level was compared to that of adherent cells.

The morphology of adherent LKB1 WT and LKB1 mutant A549 and H2030 cells and the respective tumor spheres generated from these cells are depicted in Figure 1A. In general, whereas LKB1 WT tumor spheres are tight and round‐shaped, LKB1 mutant spheres generated by A549 and H2030 cells are oval‐shaped and loose/irregular structures, respectively. To determine the role of LKB1 in regulating the self‐renewal of A549 and H2030 tumor spheres, we seeded cells into multiple wells at various cell densities (1–20 cells/well) and calculated the frequency of spheres by ELDA, a statistical method used to estimate the stem cell frequency within the tumor cell population [22]. No significant difference was observed between the frequency of tumor spheres generated by LKB1 WT A549 cells (1/1.39) versus LKB1 mutant A549 cells (1/1.47) or LKB1 WT H2030 cells (1/1.47) versus LKB1 mutant H2030 cells (1/1.62, Figure 1B).

The cell surface markers CD44 and CD133, and stem cell transcription factors such as Nanog and SOX_2,_ are often used for the identification of cancer stem cells. Assessment of the level of these proteins in adherent cells and the respective tumor spheres showed higher levels of CD44 and nanog in A549 spheres than in adherent cells, whereas levels of SOX2 and CD133 were lower in spheres (Figure 1C). In 2030 cells, both adherent cells and tumor spheres from LKB1 mutant cells exhibited a lower level of CD44 than the respective LKB1 WT cells, while levels of nanog and SOX2 were similar among the four cell groups, and expression of CD133 was inconsistent (Figure 1C). The inconsistency of CD44 levels between spheres and adherent cells of the two LKB1 mutant cell lines indicates that although tumor spheres generally exhibit higher CD44 levels, the expression can be highly heterogeneous depending on the tumor type and specific genetic background, baseline levels, and the exact CD44 isoform being measured.

LKB1 is a master upstream activator of the AMPK enzyme, which then inhibits the mTOR pathway and suppresses cell growth, proliferation, and tumor growth. Thus, LKB1 loss leads to inactivation of AMPK and increased phosphorylation of downstream mTOR targets 4E‐BP1 and S6. As depicted in Figure 1D, LKB1 loss reduced the level of phospho‐AMPK in both tumor spheres and adherent cells, the effect being stronger in tumor spheres than in adherent cells. However, unexpectedly, adherent LKB1 mutant A549 and H2030 cells and tumor spheres barely expressed phospho‐4E‐BP1, whereas LKB1 WT tumor spheres exhibited a lower level of the protein than their LKB1 WT adherent counterparts. Levels of phospho‐S6 were not different between LKB1 WT and LKB1 mutant cells, but spheres exhibited a lower level of the protein than adherent cells. Phospho‐Akt, the major upstream regulator of the mTOR pathway, and its major effector phospho‐GSK were also expressed at lower levels in LKB1 mutant and LKB1 WT A549 tumor spheres, as well as LKB1 mutant H2030 tumor spheres, when compared to the levels in the respective adherent cells.

LKB1 plays a significant role in regulating cellular oxidative stress, and therefore, loss of LKB1 function can modulate the expression of proteins involved in cellular defense against oxidative stress [23]. We assessed levels of these proteins in A549 and H2030 adherent cells and tumor spheres. Although NRF2 is a key antioxidant protein in CSCs, playing a crucial role in their survival, self‐renewal, and resistance to therapy [24], only LKB1 mutant H2030 tumor spheres expressed a higher level of the protein (Figure 1E). Also, xCT, a downstream effector of NRF2 and a protein that plays a pivotal role in GSH biosynthesis and protecting cells from oxidative stress, was markedly lower in spheres as compared with the expression in parental adherent cells, and the protein was underexpressed in LKB1 WT spheres than in LKB1 mutant spheres. On the other hand, the level of GPX4, a crucial enzyme that protects cells from oxidative damage by reducing lipid hydroperoxides using glutathione as a cofactor, was higher in tumor spheres than in adherent cells, and this difference was more evident in LKB1 WT A549 tumor spheres. Similar modulations of xCT and GPX4 were found in the other A549 clones too (Figure S1A), indicating that the observed results are not due to clonal effects but rather related to the expression of LKB1. As expected, lower xCT expression in tumor spheres, as compared to that in adherent cells, led to a lower level of both total and oxidized glutathione in tumor spheres (Figure S1B).

Given that lipid metabolism is a critical regulator of cancer stem cells and ferroptosis [25], we compared the level of acetyl‐CoA carboxylase (ACC), stearoyl‐CoA desaturase (SCD1), and acyl‐CoA synthetase long‐chain family member 4 (ACSL4) in adherent cells and tumor spheres. In line with the reduced activation of AMPK in LKB1 mutant cells, phosphorylation of ACC, which is mediated by AMPK, was lower in LKB1 mutant

adherent cells and tumor spheres, especially in the latter cells (Figure 1E). On the other hand, levels of ACSL4, which promotes the accumulation of ferroptosis‐inducing lipids such as PUFAs, and SCD1, an enzyme that protects cells from ferroptosis by increasing the formation of monounsaturated fatty acids (MUFAs), were higher in spheres, as compared to the levels in the respective adherent cells, in particular in LKB1 mutant tumor spheres.

### 3.2 LKB1 Loss Potentiated Mitomet‐Induced Inhibition of Self‐Renewal, Mitochondrial Membrane Potential (MMP), ATP Synthesis, Cell Survival/Proliferation, and Expression of Proliferation‐, Redox Balance‐, and Lipid Synthesis‐Related Proteins in NSCLC Tumor Spheres

**Figure 2:**
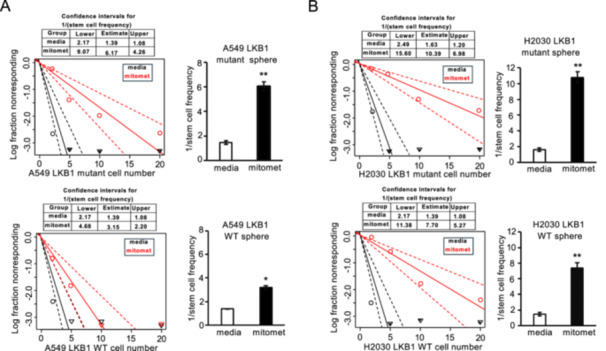
Mitomet differentially reduced the self‐renewal of LKB1 mutant A549 (A) and H2030 (B) tumor spheres. LKB1 WT and LKB1 mutant A549 and H2030 cells were cultured in sphere media, with or without 2.5 µM mitomet in a 96‐well flat‐bottom ultra‐low attachment plate, and on day 7 of culture, any wells that contained one or more spheres ≥ 20 µm in diameter were scored. Bar graphs represent the mean ± SD of three assays. *p < 0.05 and **p < 0.01 compared to cells cultured without mitomet.

Alterations in mitochondrial structure and morphology, along with the inhibition of oxidative phosphorylation (OXPHOS), are key factors in suppressing the stemness, proliferation, and survival of CSCs [26]. Therefore, we assessed if treatment with mitomet, an agent that targets mitochondria and reduces OXPHOS [27], differentially inhibits self‐renewal, mitochondrial membrane potential, ATP synthesis, cell proliferation/survival, levels of cell proliferation‐, redox balance, and lipid synthesis‐related proteins in LKB1 mutant spheres. As depicted in Figure 2A, treatment of LKB1‐mutant and LKB1 WT A549 tumor spheres with mitomet resulted in about 4.1‐fold and 2.3‐fold reduction in tumor sphere frequency, respectively, compared to untreated spheres. Mitomet also induced a stronger inhibitory effect in the self‐renewal of LKB1 mutant H2030 tumor spheres (6.7‐fold lower) than in the corresponding LKB1 WT spheres (5.0‐fold lower, 2B). Likewise, IACS‐010759, a potent OXPHOS inhibitor, significantly reduced the self‐renewal of A549 and H2030 tumor spheres, and the effects in LKB1 mutant spheres were stronger than those of LKB1 WT spheres (Figure S1C).

**Figure 3:**
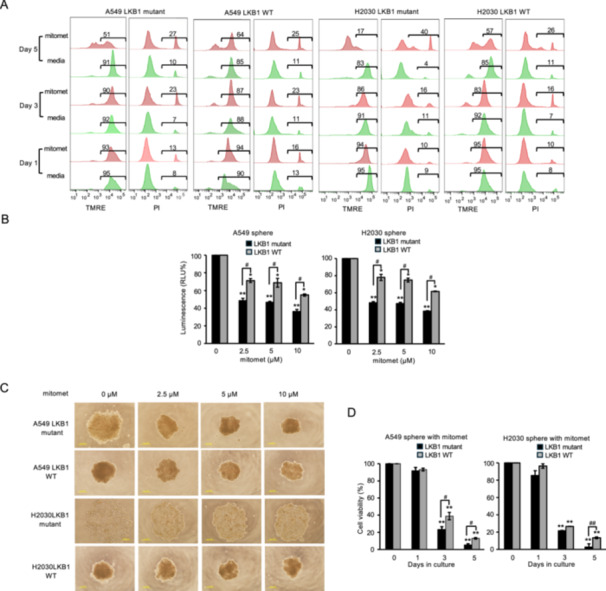
Effects of mitomet on MMP, ATP synthesis, cell proliferation, and survival of LKB1 mutant and LKB1 WT NSCLC tumor spheres. (A) Representative data showing the time‐dependent effects of mitomet on MMP and cell survival of LKB1 mutant and LKB1 WT A549 and H2030 tumor spheres. Cells were cultured in the absence or presence of mitomet (10 µM) for five days, and the number (%) of TMRE‐positive viable cells and PI‐positive dead cells was analyzed by flow cytometry. (B) Effect of mitomet on the viability of LKB1 WT and LKB1 mutant tumor spheres as assessed by measurement of ATP level on day 5 of cell culture. ATP levels were expressed as Luminescent (RLU) signal intensity. RLU intensity of each culture condition was compared to that of the media. Data represent mean ± SD of three experiments. ** *p* < 0.01 or * *p* < 0.05, compared to ATP levels in tumor spheres cultured without mitomet. ^#^ *p* < 0.05, compared to ATP level in LKB1 WT tumor spheres. (C) Morphological changes of NSCLC tumor spheres grown in the presence of different doses of mitomet for five days. Images were acquired on an Olympus IX71 inverted fluorescence microscope with an Olympus DP12 digital microscope camera at 100× magnification. (D) Kinetic changes of tumor spheres cultured with 10 µM mitomet for 5 days. The proportion of viable NSCLC cells was calculated by multiplying total cell counts from a trypan blue‐exclusion assay by the percentage of PI‐negative live cells of each culture condition. Data represent mean ± SD of three experiments. ** *p* < 0.01, compared to the number of cells cultured in the absence of mitomet; ^#^ *p* < 0.05 or ^# #^ *p* < 0.01, compared to the number of LKB1 WT tumor spheres.

To determine the potential mechanisms through which mitomet reduces the frequency of tumor spheres, we determined the effects of mitomet on mitochondrial membrane potential (MMP) and ATP synthesis, key factors in the process of oxidative phosphorylation and known to be modulated by metformin [28], the parent compound of mitomet. TMRE accumulation assay, a measurement of MMP, indicated that mitomet reduced TMRE fluorescence in a time‐dependent manner, suggesting the collapse of MMP and damage to the mitochondria (Figure 3A). These effects of mitomet were paralleled by reduced cell viability as determined by flow cytometry analysis of propidium iodide (PI)‐positive cells. In line with the evidence that mitomet reduces OXPHOS [20], ATP generation was reduced in a concentration‐dependent manner in both LKB1 mutant and LKB1 WT spheres, but the effect in LKB1 mutant spheres was significantly stronger than that in LKB1 WT cells (Figure 2B). Figure 2C depicts the effects of mitomet‐induced inhibition of ATP synthesis on the size and morphology of LKB1 WT and LKB1 mutant A549 and H2030 tumor spheres. These morphological changes were paralleled by significant and time‐dependent reductions in the viability of mitomet‐treated tumor spheres (Figure 2D).

**Figure 4:**
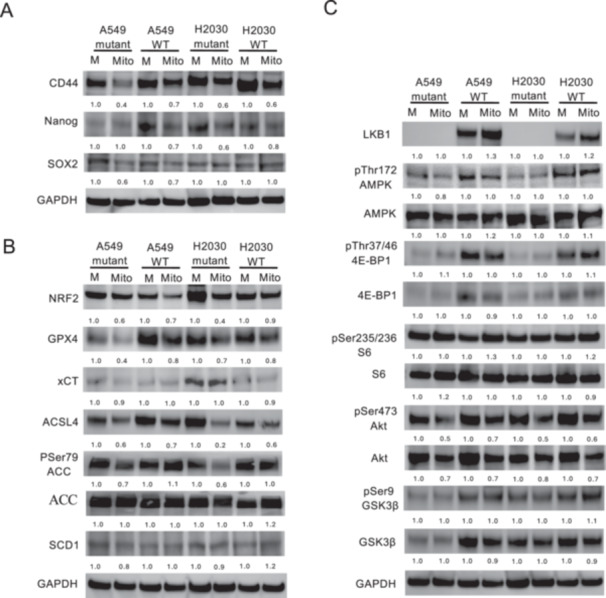
Representative Western blots showing the effect of mitomet on the level of stemness and self‐renewal‐ (A) redox and lipid synthesis, (B) cell proliferation and survival, (C) related proteins in LKB1 WT and LKB1 mutant A549 and H2030 tumor spheres. LKB1 WT and LKB1 mutant A549 and H2030 cells were cultured in sphere media in the absence and presence of mitomet for three days, cells harvested, protein lysates prepared, and Western blot assay performed as described in the Materials and Methods section. “M” and “Mito” stand for “Media” and “Mitomet” and represent tumor spheres grown in the absence or presence of mitomet, respectively. Data shown are representative results from 1 of 3 independent experiments. Protein expression was normalized to the expression of GAPDH, and relative expression of proteins in mitomet‐treated tumor spheres was compared to that of spheres grown without mitomet (media only) and indicated under each blot.

In line with its differential effects on the phenotypes of LKB1 mutant spheres, mitomet suppressed levels of CD44, p‐Akt, GPX4, NRF2, ACSL4, and p‐ACC more strongly in mutant tumor spheres than in LKB1 WT spheres (Figure 4A–C). Since CD44 and p‐Akt, the principal downstream effector of CD44, are critical regulators of stemness, the preferential effects of mitomet on the self‐renewal of LKB1 mutant spheres could be ascribed, at least in part, to the downregulation of these proteins. To prove if GPX4 protein stability is reduced under mitomet treatment, we performed a cycloheximide (CHX) chase assay. Compared to spheres treated with CHX only, spheres treated with CHX and mitomet exhibited an increase in the degradation rate of GPX4 (Figure S1D), and these effects of mitomet were suppressed by MG132, a potent proteasome inhibitor. These results suggest that mitomet‐induced degradation of GPX4 might be mediated via the ubiquitin–proteasome pathway. Unexpectedly, mitomet did not modulate levels of xCT (Figure 4B) and glutathione (Figure S1B) in tumor spheres, which suggests that mitomet‐induced ferroptosis may be mediated via a glutathione‐independent mechanism.

### 3.3 Mitomet Differentially Increased Mitochondrial Reactive Oxygen Species (mROS) and Lipid Peroxidation Levels in LKB1 Mutant NSCLC Tumor Spheres

**Figure 5:**
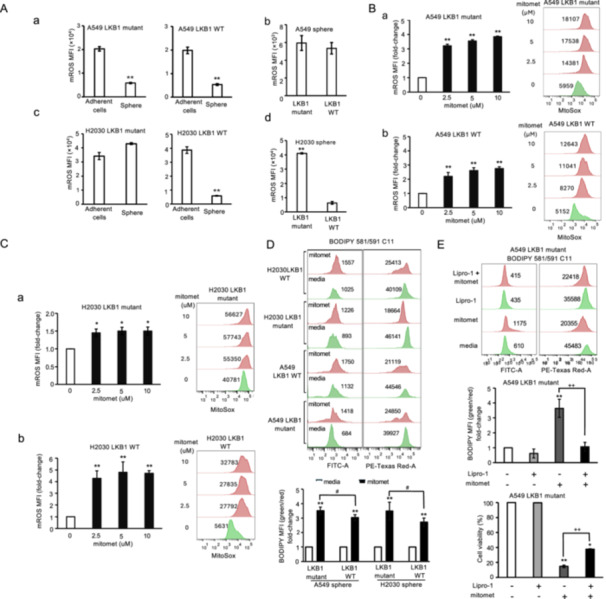
Background and mitomet‐induced mROS levels and lipid peroxidation in NSCLC tumor spheres. (A) Levels of background mROS in LKB1 WT and LKB1 mutant A549 and H2030 adherent cells and the respective tumor spheres as determined by flow cytometry after staining single cells with MitoSox Red Mitochondrial Superoxide Indicator. Data shown are the mean ± SD of mROS level from three experiments. *p < 0.05, compared to mROS level in adherent cells (a, c) or LKB1 WT spheres (b, d). (B, C) Effect of mitomet on mROS level in A549 (B) and H2030 (C) tumor spheres treated with the drug for three days. The depicted histograms are one of the three representative results and bar graphs show mean ± SD of mROS MFI from three experiments. *p < 0.05 or **p < 0.01, compared to mROS MFI from spheres grown without mitomet. (D) Induction of lipid peroxidation by mitomet. Tumor spheres were treated with mitomet (10 µM) for five days, and the level of lipid peroxidation was determined by flow cytometry (reduced state at the 581/591 nm and oxidation state at 488/510 nm) after staining spheres with BODIPY 581/591 C11. Data shows representative histograms and mean ± SD of MFI BODIPY (green/red) from three experiments. *p < 0.01, compared to cells grown without mitomet or compared to LKB1 WT mutant tumor spheres. ^#^ *p* < 0.05, compared to the effect in LKB1 WT tumor spheres. (E) The antioxidant lipro‐1 significantly suppressed mitomet‐induced lipid peroxidation and antiproliferative effects in tumor spheres. Spheres were treated with mitomet alone (10 µM), lipro‐1 alone, or a combination of mitomet and lipro‐1 and lipid peroxidation and cell proliferation were determined on day 5 or 3, respectively. Data show representative flow cytometry histograms for lipid peroxidation and mean ± SD of MFI BODIPY and cell proliferation from three experiments. *p < 0.05 and ** *p* < 0.01, as compared to cells not treated with mitomet or treated with mitomet alone; ^++^ *p* < 0.01, compared to the effect in LKB1 WT tumor spheres.

LKB1 plays a crucial role in maintaining redox homeostasis and activating pathways that counteract oxidative stress [23]. Therefore, LKB1 deficiency typically leads to elevated basal levels of mROS in NSCLC cells [19]. However, tumor spheres generated by LKB1 mutant A549 cells have been found to exhibit lower intracellular ROS levels compared to the respective adherent cells [7]. In the present study, where we compared mROS levels in adherent cells and tumor spheres, tumor spheres exhibited much lower mROS levels than the respective adherent cells, except for H2030 mutant spheres, which have an inherently high level of mROS (Figures 5A‐a and 5A‐c). Among the tumor spheres, LKB1 mutant tumor spheres from both A549 and H2030 cells exhibited higher mROS levels than LKB1 WT spheres, although the difference between A549 spheres was not significant (Figures 5A‐b and 5A‐d). Mitomet significantly increased mROS level in both LKB1 mutant and LKB1 WT A549 tumor spheres, and the effects in LKB1 mutant A549 spheres were markedly higher than the respective LKB1 WT spheres (Figures 5B‐a, 5B‐b). Similarly, mitomet‐treated H2030 spheres exhibited significant increases in mROS levels, but, due to the higher background level of mROS in LKB1 mutant spheres than in LKB1 WT spheres, the effect of the drug in LKB1 WT spheres appeared stronger (Figures 5C‐a, 5C‐b), although absolute mROS levels were much higher in mutant spheres.

High levels of mROS induce adverse effects on cell components, including DNA, proteins, and lipids. Due to their high level of polyunsaturated fatty acids, cellular membranes or organelle membranes are particularly vulnerable to mROS‐induced damage, often known as lipid peroxidation [29]. Therefore, we used BODIPY™ 581/591 C11 (lipid peroxidation sensor), a fluorescent dye that localizes to membranes and measures lipid peroxidation in live cells. Unlike mROS, background lipid peroxidation level was not different between LKB1 mutant and LKB1 WT A549 and H2030 tumor spheres (data not shown). However, upon mitomet treatment, lipid peroxidation level was significantly increased in both LKB1 WT and LKB1 mutant A549 and H2030 spheres. The effect of mitomet was significantly stronger in LKB1 mutant spheres than in LKB1 WT spheres in both cell lines (Figure 5D). Given that mitomet induced mROS, reduced levels of GPX4, a key defense against lipid peroxidation, and increased lipid peroxidation, all of which are important features of ferroptosis, we postulated that mitomet‐induced cell death in tumor spheres could be mediated via ferroptosis. Indeed, co‐treatment of A549 tumor spheres with mitomet and Lipro‐1, a potent and selective inhibitor of ferroptosis that inhibits cell death by restoring the expression of GPX4, significantly reduced mitomet‐induced lipid peroxidation and anti‐proliferative effects (Figure 5E). Similar results were found in H2030 spheres (Figure S1E). On the other hand, co‐treatment of tumor spheres with mitomet and the pan‐caspase inhibitor z‐VAD‐FMK did not modulate the effect of mitomet (data not shown).

### 3.4 Silencing of GPX4 with a Specific siRNA Reduced the Proliferation of Tumor Spheres

**Figure 6:**
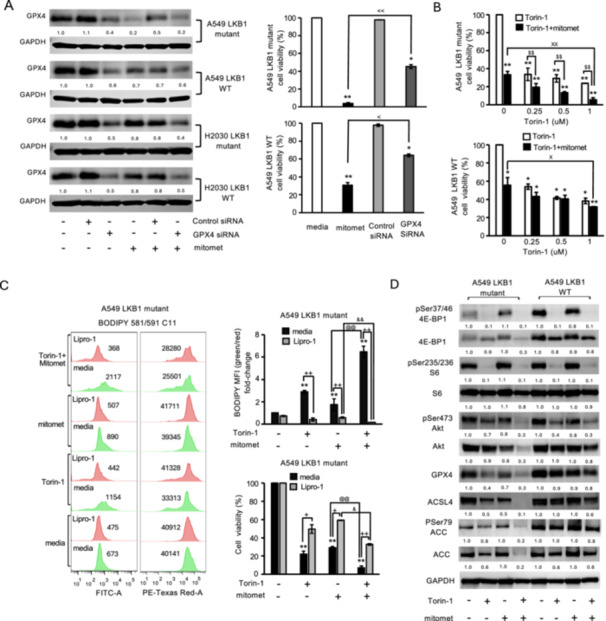
Effects of GPX4 silencing or mTOR inhibition on tumor spheres. (A) Comparative effects of GPX4 siRNA and mitomet on GPX4 expression and tumor sphere growth. NSCLC cells were cultured in sphere media with or without siRNA, mitomet, or siRNA+mitomet for three days and expression of GPX4 and cell viability were determined by Western immunoblotting and trypan blue exclusion assay, respectively. Data shown are representative immunoblot of GPX4 and mean ± SD of cell viability from 3 individual experiments. GPX4 level was normalized to the expression of GAPDH and relative expression was compared to that of the media and indicated under each blot. **p < 0.01 or *p < 0.05, compared to the viability of untreated spheres. (B) The mTOR inhibitor Torin‐1 potentiated mitomet‐induced toxicity in tumor spheres. Bar graphs show the viability of LKB1 mutant and LKB1 WT A549 tumor spheres treated with different doses of Torin‐1 in the absence or presence of mitomet (2.5 µM) for 3 days. Data are mean ± SD from 3 individual experiments. **p < 0.01 or *p < 0.05, compared to the viability of untreated spheres. ^$$^ *p* < 0.01, compared to spheres treated with Torin‐1 alone; ^xx^ *p* < 0.01, compared to spheres treated with mitomet alone. (C) Lipro‐1 suppressed lipid peroxidation and cell death induced by mitomet + Torin‐1 in A549 LKB1 mutant tumor spheres treated with the drugs for five days. Histograms represent one of three flow cytometry‐based studies in which MFI of BODIPY 581/591 C11 oxidation state (Green color, FITC channel) and reduced state (Red color, PE‐Texas channel) were determined. Cell viability was assessed by the trypan blue exclusion assay. Bar graphs are mean ± SD from three experiments showing Torin‐1‐, mitomet‐, or Torin‐1+mitomet‐induced lipid peroxidation or cell death in tumor spheres and suppression of these effects by Lipro‐1. **p < 0.01, compared to untreated spheres. ^+^ *p* < 0.05, ^++^ *p* < 0.01, compared to spheres not treated with Lipro‐1, ^@@^ *p* < 0.01, effect in mitomet+Torin‐1‐treated cells compared to cells treated with mitomet alone in the absence of Lipro‐1; ^&^ *p* < 0.05, ^&^ ^&^ *p* < 0.01, effect in mitomet+Torin‐1‐treated cells compared to cells treated with mitomet alone in the presence of Lipro‐1. (D) Representative Western blots showing the effect of Torin‐1 and/or mitomet on the expression of mTOR‐Akt‐GPX4 pathway‐related proteins in LKB1 mutant A549 tumor spheres. Protein expression was normalized to the expression of GAPDH, and the relative expression was compared to that of the media and indicated under each blot.

Our foregoing findings showed that GPX4 is overexpressed in A549 and H2030 tumor spheres, and its level was markedly reduced by mitomet, suggesting that mitomet‐induced ferroptosis in these tumor spheres could be mediated, at least in part, via suppression of GPX4 levels. To elucidate the role of GPX4 depletion in mitomet‐induced ferroptosis in tumor spheres, LKB1 WT and LKB1 mutant spheres were treated with siRNA specific to GPX4, control siRNA, mitomet, or a combination of mitomet and siRNA. The effects on GPX4 protein levels and sphere proliferation were then determined. As shown in Figure 6A, both GPX4 siRNA and mitomet reduced the level of GPX4 protein in A549 and H2030 tumor spheres irrespective of LKB1 expression status. However, apart from mitomet‐treated LKB1 mutant A549 cells in which the effect of mitomet was stronger than that of GPX4 siRNA, GPX4 siRNA was more potent than mitomet in reducing the level of GPX4, but failed to potentiate the effects of mitomet on GPX4. Despite the overall weaker effect of mitomet compared to GPX4 siRNA in suppressing GPX4 levels, the antiproliferative effects of mitomet were stronger than those of GPX4 siRNA. These results suggest that inhibition of GPX4 may not be the only mechanism through which mitomet induces ferroptosis in tumor spheres.

### 3.5 Targeting mTOR Potentiated Mitomet‐Induced Lipid Peroxidation and Ferroptotic Effects

Given the reports that mutation of PI3K‐AKT‐mTOR signaling protects cancer cells from oxidative stress and ferroptotic death and inhibition of this pathway potentiates the cancer therapeutic effect of ferroptosis inducers [30, 31], we sought to determine if mTOR inhibition also potentiates mitomet‐induced ferroptosis. Indeed, co‐treatment of tumor spheres with mitomet and the mTOR catalytic inhibitor Torin‐1 differentially increased the cytotoxicity of mitomet to tumor spheres derived from LKB1 mutant A549 (Figure 6B) and H2030 (Figure S1F) cells. These effects were associated with increased lipid peroxidation, and Lipro‐1, a small molecule inhibitor of ferroptosis, significantly suppressed mitomet+torin‐induced lipid peroxidation and ferroptosis in A549 LKB1 mutant spheres (Figure 6C). In agreement with the results in cytotoxicity and lipid peroxidation assays, Torin‐1 differentially enhanced mitomet‐induced suppression of GPX4, ACSL4, and phospho‐ACC in LKB1 mutant tumor spheres (Figure 6D). However, levels of phospho‐4E‐BP1, phospho‐S6, and phospho‐Akt were regulated in the same manner in both LKB1 WT and LKB1 mutant tumor spheres.

## 4 Discussion

LKB1mutant tumors exhibit a subset of cells with stemness properties that typically display enhanced malignant and metastatic potential, chemoresistance, and tumor recurrence [32, 33]. Therefore, one promising approach for the treatment of this group of cancers could be targeting CSCs. At present, CSCs are targeted with antibodies directed to CSC‐specific surface markers, transcription factors, and signaling pathways. However, targeting these biomarkers is beset with problems since they are heterogeneous and non‐specific, and exhibit a high degree of plasticity, which can cause instability of the CSC phenotype and reversion of their expression [33]. Moreover, CSC markers, transcription factors, and signaling pathways are shared with normal stem cells, raising concerns about potential off‐target effects and toxicity to healthy tissues. Alternative strategies to eliminate lung CSCs could be targeting vulnerabilities related to energy metabolism and antioxidant defense systems [7]. These vulnerabilities are particularly relevant to target LKB1 mutant lung CSCs, as these cells are unable to sufficiently upregulate AMPK‐mediated glycolysis and depend heavily on OXPHOS [6]. Moreover, disruption in the LKB1‐AMPK signaling pathway, an important negative regulator of ferroptosis [34], makes cells more susceptible to ferroptosis. Thus, OXPHOS inhibitors, whose key mechanisms of action are targeting mitochondrial energy production and induction of ROS production, could selectively disrupt the redox balance and induce cell death in LKB1 mutant CSCs, which are known not to tolerate high levels of ROS [35]. In the present study, we used isogenic pairs of LKB1 mutant/WT NSCLC tumor spheres to determine if LKB1 mutation status is associated with increased sensitivity to mitomet, a potent inhibitor of complex I of OXPHOS and inducer of ROS within cancer cell mitochondria. LKB1 mutant tumor spheres, compared to LKB1 WT tumor spheres, exhibited a lower level of endogenous GPX4 and a higher level of mROS. Mitomet further reduced GPX4 in LKB1 mutant speres, leading to a differential increase in mROS, lipid peroxidation, and ferroptosis. Interestingly, Torin‐1, a highly potent and selective inhibitor of both mTOR1 and mTOR2 complexes, differentially potentiated mitomet‐induced modulation of GPX4 and lipid peroxidation in LKB1 mutant tumor spheres and sensitized these cells to mitomet‐induced antiproliferative effects and ferroptosis. These results indicate that the PI3K‐AKT‐mTOR signaling pathway protects LKB1 mutant tumor spheres from oxidative stress and ferroptotic cell death, and targeting mTOR potentiates the ferroptotic effects of mitomet.

Among the five OXPHOS complexes that are involved in ATP synthesis, complex I plays the most significant role in modulating ferroptosis [36] as it mediates the production of reduced coenzyme Q (CoQH_2_, ubiquinol), a radical‐trapping antioxidant capable of effectively suppressing ferroptosis [37]. Thus, inhibition of complex I could deplete CoQH2 and generate superoxide radicals possessing the ability to induce lipid peroxidation and ferroptosis in cancer cells. However, currently, there is no safe and potent inhibitor of complex I. The antidiabetic drug metformin, the parent drug of mitomet, is well‐tolerated and inhibits complex I and tumor cell mitochondrial respiration, but its effects on the mitochondria are weak [38] and low potency [19, 20]. Although IACS‐010759, a clinical‐grade small‐molecule inhibitor of complex I, induces significant energy depletion in cancer cells, it exerts unacceptable dose‐limiting toxicity [39]. Our findings suggest that mitomet could be superior to metformin or IACS‐010759 for the treatment of LKB1 mutant NSCLC owing to its potent inhibition of mitochondrial complex I and selective toxicity towards cancer cells [19, 20, 21], probably due to the higher mitochondrial membrane potential and uptake of the drug by cancer cells as compared to normal cells [40].

The LKB1‐AMPK pathway is an important regulator of ferroptosis [16, 17]. Specifically, this pathway acts as a negative regulator of ferroptosis by inducing phosphorylation‐mediated inactivation of acetyl‐CoA carboxylase (ACC [16, 17]), a key enzyme in the synthesis of fatty acids and required for the initiation of ferroptosis. Genetic knockout of AMPK or LKB1 genes or mutation of the LKB1 gene in mouse and human cell lines reduced ACC phosphorylation, increased levels of PUFAs, and sensitivity to ferroptosis‐inducing agents [16, 17, 34]. On the other hand, LKB1 mutation, particularly in conjunction with KEAP1 loss, has been shown to elevate expression of ferroptosis‐protective genes, including cystine/glutamine antiporter xCT, stearoyl‐CoA desaturase 1 (SCD1), and aldo‐keto‐reductase family proteins, thereby increasing the cellular threshold of NSCLC cells to ferroptosis [41, 42]. The reasons for these discrepant findings are unclear. Our results are in line with the reports by Lee et al. [16] and Li et al. [17] since LKB1 and KEAP1 mutant NSCLC tumor spheres exhibited differential sensitivity to mitomet‐induced ferroptosis, and xCT expression was significantly lower in tumor spheres and proved dispensable for mitomet‐induced ferroptosis

Ferroptosis is also regulated by the PI3K‐AKT‐mTOR signaling pathway through various mechanisms, including modulation of lipid metabolism and redox homeostasis. When LKB1 is mutated or lost, and AMPK activation is reduced, the inhibitory signals to the mTOR pathway are weakened. This results in increased mTOR activity and ferroptosis resistance through mTORC1‐phospho‐S6‐dependent induction of sterol regulatory element‐binding protein 1 (SREBP1)‐SCD1 pathway, which increases the ratio of MUFAs to PUFAs and makes cell membranes less vulnerable to lipid peroxidation and ferroptosis [30]. SCD1, a protein that reduces the susceptibility of cancer cells to ferroptosis, has been consistently found to be overexpressed in CSCs across various cancer types, including lung cancer [43, 44], contributing to their self‐renewal, resistance to chemotherapy, and the overall progression of the disease. However, in our studies, there was no correlation between the expression of phospho‐S6 and SCD1 in tumor spheres, and mitomet modulated neither the level of phospho‐S6/total S6 nor SCD1, suggesting that the mTOR‐SREBP1‐SCD1 pathway is probably dispensable for mitomet‐induced ferroptosis in these cells.

Another mechanism connecting mTORC1 and ferroptosis is cyst(e)ine‐mediated activation of mTORC1‐4E‐BP1, through Rag GTPases, which subsequently promotes the synthesis of GPX4 [31], thereby preventing the initiation and progression of ferroptosis. In the present study, we found that, as compared to the level in LKB1 WT spheres and adherent cells, phospho‐4E‐BP1 was markedly underexpressed in LKB1 mutant spheres and adherent cells. Moreover, mitomet markedly reduced the level of GPX4 in LKB1 mutant spheres without modulating the expression of phospho‐4E‐BP. Together, our data show that the mTOR pathway is not involved in mitomet‐induced ferroptosis in LKB1 mutant NSCLC spheres and suggest that targeting the mTOR pathway may potentiate ferroptotic activities of mitomet in LKB1 mutant tumor spheres. Indeed, inhibition of mTOR by its catalytic inhibitor Torin differentially and significantly sensitized LKB1 mutant tumor spheres to mitomet‐induced lipid peroxidation and ferroptosis. These effects were markedly inhibited by the ferroptosis inhibitor Lipro‐1. These results are in line with previous reports, which showed that pharmacological or genetic inactivation of mTORC1 decreased GPX4 protein levels and sensitized cancer cells to ferroptosis and synergized with ferroptosis inducers to suppress tumor growth in vivo [30, 31]. However, although silencing of GPX4 with a specific siRNA reduced the expression of the GPX4 protein as strongly as mitomet, inhibition of sphere growth by GPX siRNA was significantly less than that of mitomet, suggesting that inhibition of GPX4 may not be the only mechanism through which mitomet induces ferroptosis.

In conclusion, we showed that mitomet differentially increased the generation of mROS in LKB1 mutant NSCLC tumor spheres, and the resulting oxidative damage led to a decline in MMP, ATP, and GPX4 and an increase in lipid peroxidation, which then culminated in ferroptosis. Furthermore, the mTOR inhibitor Torin 1 potentiated the ferroptotic activities of mitomet, which could be related to suppression of LKB1 mutation‐associated derepression of the mTOR pathway and protection of CSCs from oxidative damage and ferroptotic cell death. Therefore, a combination of mitomet and mTOR inhibitors could be a promising approach for the prevention and treatment of LKB1 mutant NSCLC.

## Author Contributions

Fekadu Kassie conceptualized, designed, and supervised the study, wrote the initial draft, and obtained funding for the research. Xueqing Liang performed all the experiments and contributed to the write‐up of the manuscript. Gunda I. Georg, Peter Dosa, and Ali Nakhi synthesized mitomet and contributed to the write‐up and final approval of the manuscript.

## Ethics Statement

The authors have nothing to report.

## Conflicts of Interest

The authors declare that they have no conflicts of interest.

## Supporting information


**Figure S1:** (A) Representative Western blots showing GPX4 and xCT protein levels in LKB1 mutant A549 cells. LKB1 overexpressing LKB1 mutant cells were cultured in tumor sphere media in 6‐well ultra‐low attachment plate for 24 hours and harvested for western blot. Data shown are representative results from 1 of 3 independent experiments. Protein expression was normalized to the expression of GAPDH and relative expression of proteins (number under the blot) was shown. (B) Measurement of intracellular GSH in mitomet‐treated LKB1 mutant and LKB1 WT A549 adherent cells and tumor spheres treated with/without 10 µM mitomet for 3 days. Data are presented as mean ± SD from 3 experiments. *p < 0.05, compared to untreated adherent cells. (C) IACS‐010759 differentially reduced the self‐renewal of LKB1 mutant/WT A549 tumorspheres. LKB1 WT and mutant A549 cells were cultured in sphere media, with or without 10 µM IACS‐010759 in 96‐well flat bottom ultra‐low attachment plate. On day 7 of cultures, any wells that contain one or more spheres (≥ 20 µm diameter) were scored. Data were analyzed using the ELDA software. Bar graphs represent mean ± SD of 3 assays. **p < 0.01, compared to cultures without IACS‐010759. (D) Effects of cycloheximide (CHX) and/or MG132 on GPX4 protein expression. (a) Western blot results showing GPX4 expression in A549 LKB1 mutant adherent cells cultured with or without 10 µM mitomet for 24 h, and then treated with 100 µg/ml CHX for 0, 8, 16, and 24 h. (b) Western blot results of A549 LKB1 mutant adherent cells cultured with or without 10 µM mitomet for 24 h and then cultured for additional 24 h in the presence or absence of 100 µg/ml CHX with or without 10 µM MG132. Data shown are representative results from 1 of 3 independent experiments. Protein expression was normalized to the expression of GAPDH and relative expression of proteins (number under the blot) was compared to 0 h media (a) or untreated cells (b). (E) The antioxidant lipro‐1 significantly suppressed mitomet‐induced anti‐proliferative effects in H2030 LKB1 mutant tumor spheres. Bar graphs show the viability of H2030 LKB1 mutant spheres treated with mitomet alone (10 µM), lipro‐1 alone or combination of mitomet and lipro‐1 for 3 days. Data are mean ± SD from 3 individual experiments. **p < 0.01 or *p < 0.05, compared to untreated spheres; ^++^ *p* < 0.01, compared to spheres treated with mitomet alone. (F) The mTOR inhibitor Torin‐1 potentiated mitomet‐induced toxicity in tumor spheres. Bar graphs show the viability of LKB1 mutant and LKB1 WT H2030 tumor spheres treated with different doses of Torin‐1 in the absence or presence of mitomet (2.5 µM) for 3 days. Data are mean ± SD from 3 individual experiments. **p < 0.01 or *p < 0.05, compared to untreated spheres. ^$^ *p* < 0.05, compared to spheres treated with Torin‐1 alone; ^x^ *p* < 0.05, compared to spheres treated with mitomet alone.

## Data Availability

The data that support the findings of this study are available on request from the corresponding author. The data are not publicly available due to privacy or ethical restrictions.
