# Supplementary figures and images for: LKB1 Loss Sensitizes Lung Tumor Spheres to Mitomet‐Induced Ferroptosis, and These Effects are Enhanced by mTOR Inhibition
Source: Mol Carcinog. 2026 May 15;65(8):963–76. doi: 10.1002/mc.70129 (PMC13372401; doi:10.1002/mc.70129)

Supplemental Figure 1

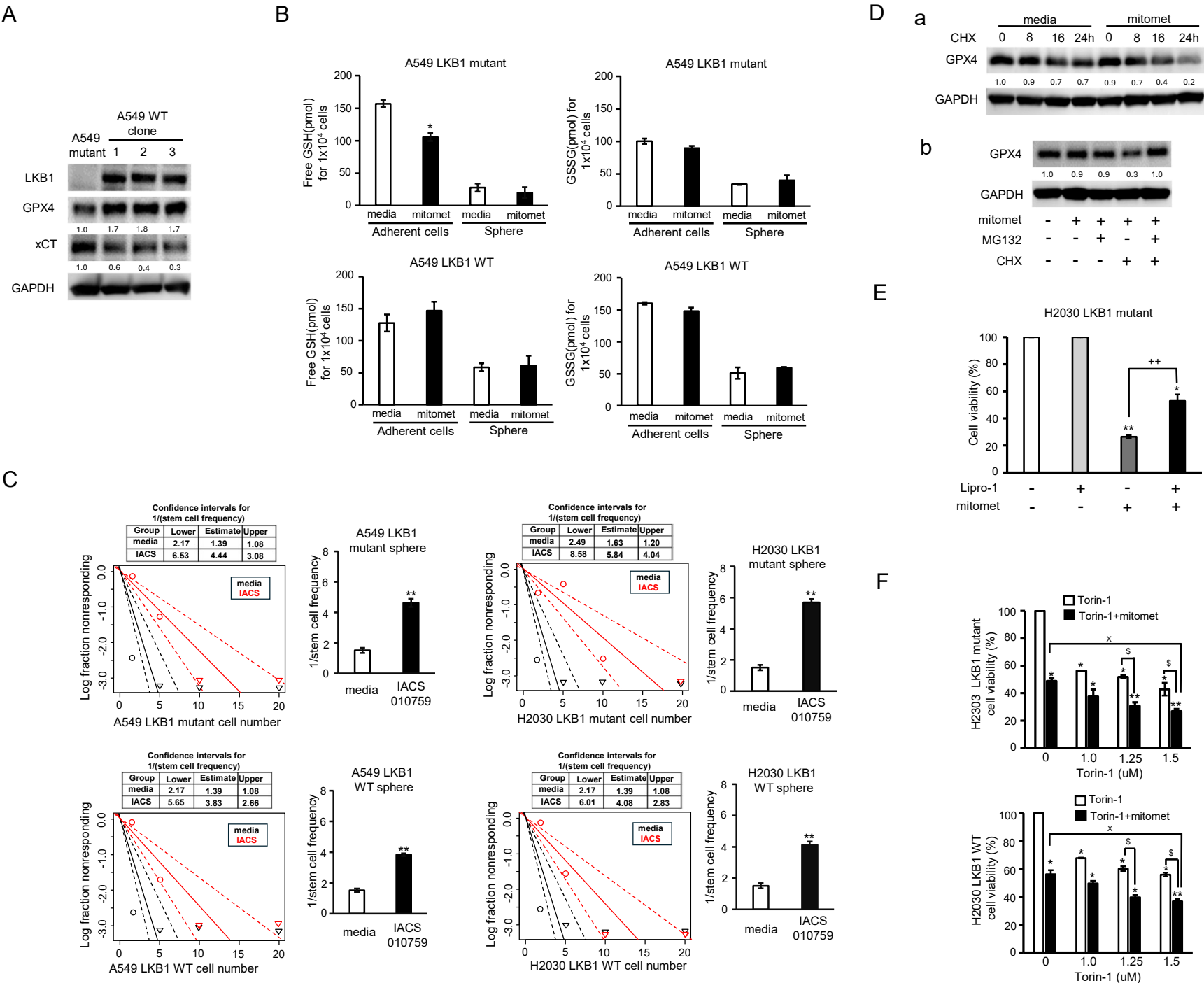

Supplement: Supplementary file 1 — Figure S1: (A) Representative Western blots showing GPX4 and xCT protein levels in LKB1 mutant A549 cells. LKB1 overexpressing LKB1 mutant cells were cultured in tumor sphere media in 6‐well ultra‐low attachment plate for 24 hours and harvested for western blot. Data shown are representative results from 1 of 3 independent experiments. Protein expression was normalized to the expression of GAPDH and relative expression of proteins (number under the blot) was shown. (B) Measurement of intracellular GSH in mitomet‐treated LKB1 mutant and LKB1 WT A549 adherent cells and tumor spheres treated with/without 10 µM mitomet for 3 days. Data are presented as mean ± SD from 3 experiments. *p < 0.05, compared to untreated adherent cells. (C) IACS‐010759 differentially reduced the self‐renewal of LKB1 mutant/WT A549 tumorspheres. LKB1 WT and mutant A549 cells were cultured in sphere media, with or without 10 µM IACS‐010759 in 96‐well flat bottom ultra‐low attachment plate. On day 7 of cultures, any wells that contain one or more spheres (≥ 20 µm diameter) were scored. Data were analyzed using the ELDA software. Bar graphs represent mean ± SD of 3 assays. **p < 0.01, compared to cultures without IACS‐010759. (D) Effects of cycloheximide (CHX) and/or MG132 on GPX4 protein expression. (a) Western blot results showing GPX4 expression in A549 LKB1 mutant adherent cells cultured with or without 10 µM mitomet for 24 h, and then treated with 100 µg/ml CHX for 0, 8, 16, and 24 h. (b) Western blot results of A549 LKB1 mutant adherent cells cultured with or without 10 µM mitomet for 24 h and then cultured for additional 24 h in the presence or absence of 100 µg/ml CHX with or without 10 µM MG132. Data shown are representative results from 1 of 3 independent experiments. Protein expression was normalized to the expression of GAPDH and relative expression of proteins (number under the blot) was compared to 0 h media (a) or untreated cells (b). (E) The antioxidant lipro‐1 significantly suppre [file MC-65-963-s001.pdf]
